# Comparison of osseointegration in areas grafted with different osteoconductive biomaterials. Preclinical study

**DOI:** 10.1590/0103-6440202204378

**Published:** 2022-03-07

**Authors:** Vithor Xavier Resende de Oliveira, Felipe Eduardo Pinotti, Rosemary Adriana Chiérici Marcantonio, Elcio Marcantonio, Guilherme José Pimentel Lopes de Oliveira

**Affiliations:** 1 Department of Periodontology/ Implantodontology, School of Dentistry, Universidade Federal de Uberlândia(FOUFU), Uberlândia, Brazil.; 2 Department of Diagnosis and Surgery, School of Dentistry at Araraquara(FOAr-Unesp), Araraquara, Brazil.

**Keywords:** Bone substitutes, dental implants, osseointegration

## Abstract

This study evaluated osseointegration in areas grafted with deproteinized bovine bone (DBB) and biphasic ceramic based on hydroxyapatite and beta-tricalcium phosphate (HA/TCP) in rat tibias. Noncritical bone defects were made in the tibias of 28 rats that were randomly assigned to 2 groups: DBB: DBB-filled defects and HA/TCP: HA/TCP-filled defects. Bone defects were made in the tibias bilaterally and filled with biomaterials. After 60 days, the implants were inserted, and the animals were euthanized 15 and 45 days after the implants were installed. Osseointegration was evaluated by biomechanical, microtomographic and histometric analysis. Implants installed in the defects filled with DBB presented higher removal torque forces (2.33 ± 0.51 Ncm vs. 1.50 ± 0.54 Ncm) and mineralized tissue volume around implants at 15 days (34.96 ± 3.68 % vs. 25.61 ± 2.95 %) and greater bone-implant contact (20.87 ± 8.28 % vs. 11.52 ± 7.42 %) and bone area within implant threads (26.83 ± 12.35 % vs. 11.98 ± 7.56 %) at 45 days compared to the measurements of implants in areas grafted with HA/TCP. Implants installed in defects in areas grafted with DBB had a better osseointegration pattern than implants placed in defects in areas grafted with HA/TCP.

## Resumo

Este estudo avaliou a osseointegração em áreas enxertadas com osso bovino desproteinizado (DBB) e cerâmica bifásica à base de hidroxiapatita e beta-fosfato tricálcico (HA / TCP) em tíbias de ratos. Defeitos ósseos não críticos foram feitos nas tíbias de 28 ratos que foram divididos aleatoriamente em 2 grupos: DBB: defeitos preenchidos com DBB e HA / TCP: defeitos preenchidos com HA / TCP. Defeitos ósseos foram confeccionados nas tíbias bilateralmente e preenchidos com biomateriais. Após 60 dias, os implantes foram instalados e os animais sacrificados 15 e 45 dias após a instalação dos implantes. A osseointegração foi avaliada por análises biomecânica, microtomográfica e histométrica. Os implantes instalados nos defeitos preenchidos com DBB apresentaram maiores forças de torque de remoção (2,33 ± 0,51 Ncm vs. 1,50 ± 0,54 Ncm) e volume de tecido mineralizado ao redor dos implantes aos 15 dias (34,96 ± 3,68% vs. 25,61 ± 2,95%) e maior contato osso-implante (20,87 ± 8,28% vs. 11,52 ± 7,42%) e área de osso dentro das roscas do implante (26,83 ± 12,35% vs. 11,98 ± 7,56%) no período de 45 dias em comparação com os implantes em áreas enxertadas com HA / TCP. Implantes instalados em áreas enxertadas com DBB apresentaram melhor padrão de osseointegração do que implantes colocados em áreas enxertadas com HA / TCP.

## Introduction

Technological advances in dental implant macrostructure and microstructure associated with improvements in surgical techniques have allowed the use of dental implants to treat all types of edentulism ^1^ ^,^ ^2^. However, areas with limited bone availability do not allow direct implant placement in appropriate positions ^3^. Therefore, the use of bone substitutes has been proposed as an alternative for case resolution of sites with a limited amount of bone for implant placement ^4^ ^,^ ^5^. Among these biomaterials, autogenous bone grafts are considered the gold standard since they are unique biomaterials that present the biological properties of osteoconduction, osteoinduction and osteogenesis bone formation ^6^ ^,^ ^7^. However, the limitations of this approach have stimulated the use of bone substitutes from other sources, such as xenogeny and alloplastic biomaterials ^8^.

Bone substitute alternatives to autogenous bone grafts have shown good clinical results in increasing the bone availability (5,6) and success of implants installed in these areas ^9^ ^,^ ^10^. Among these biomaterials, the use of deproteinized bovine bone (DBB) and biphasic ceramic based on hydroxyapatite and beta-tricalcium phosphate (HA/TCP) deserves to be highlighted since these biomaterials presented good implant success rates in clinical studies ^5^ ^,^ ^9^ ^,^ ^10^. However, since these biomaterials only present bone formation properties by osteoconduction, they have a delayed bone repair process compared to autogenous bone grafts ^6^ ^,^ ^11^ and reduce the survival rates of dental implants placed in these areas compared with implants placed in native bone ^5^. Furthermore, there are still doubts as to the best moment of load installation in implants placed in grafted areas with these different biomaterials, because despite comparisons of the quality of the grafted areas, the evaluations were conducted previously at the time of implant installation ^10^ ^,^ ^11^ ^,^ ^12^, and the osseointegration process in these areas has not been thoroughly investigated ^13^ ^,^ ^14^. Thus, the objective of this study was to compare osseointegration in areas grafted with DBB and with HA/TCP in a preclinical model of rat tibia.

## Methodology

This study was submitted and approved by the Animal Ethics Committee of Universidade Federal de Uberlândia (CEUA: 11/2020). Twenty-eight rats (*Rattus norvegicus*, Holtzman variation), 12 weeks old, weighing 250-300 g, were used for this study. The animals were kept in an environment with temperature (21 ± 1°C), humidity (65-70%), and controlled light cycles (12 hours). The animals were offered water and *feed ad libitum.* This study was conducted according to the ARRIVE protocol for conducting preclinical studies.

### Groups

The animals were randomly assigned to 2 groups of 14 animals each, which were divided according to the type of biomaterial that was used to fill the bone defects. The two groups were the DBB group: defect filled with deproteinized bovine bone (Bio-Oss®, Geistlich AG, Wolhusen, Switzerland) and the HA/TCP Group: defect filled with beta-tricalcium phosphate/Hydroxyapatite (Straumann® Bone Ceramic, Straumann AG, Basel, Switzerland). A surface-machined implant was placed in the bone defects in both groups (Neodent®, Curitiba, PR, Brazil). Each cage allocated 3-4 four animals and the randomization were performed by lot, separating each cage for a specific group using the random.org site.

### Surgical procedure

The animals were anesthetized by a combination of ketamine (Agener União Ltda, Sao Paulo, SP, Brazil) at a dosage of 0.08 ml/100 g body mass with xylazine (Rompum, Bayer SA, Sao Paulo, SP, Brazil) at a dosage of 0.04 ml/100 g body mass. Subsequently, the animals underwent a trichotomy of the internal region of the right and left hind paws, and antisepsis was performed.

An approximately 10-mm incision was made in planes over the tibial tuberosity. After delicate dissection, the bone tissue was submitted to osteotomy by means of a countermounted spherical drill with the aid of a 1200 rpm electric motor (BLM 600 - Driller, São Paulo, SP, Brazil) under abundant solution irrigation with sterile saline. Each defect that was formed had final measurements of 4 mm in length and width and 1.5 mm in depth; defects were later filled with biomaterials. The defects were measured with the aid of a periodontal probe (Millenium, Golgran, São Caetano do Sul, SP, Brazil). The tissue was sutured by planes internally with 5.0 resorbable thread (Vicryl Ethicon, Johnson & Johnson, Sao Jose dos Campos, Brazil) and externally with 4.0 silk thread (Ethicon, Johnson & Johnson, Sao Jose dos Campos, Brazil). The animals received a single dose of streptomycin-associated penicillin at a dosage of 0.1 ml/kg (Multibiotic Small, Vitalfarma, Sao Sebastiao do Paraíso, MG, Brazil) and 0.1 ml/kg ketoprofen (Ketoflex; Mundo Animal, São Paulo, Brazil) intramuscularly.

After a period of 60 days, a second surgical intervention was performed in the region that received the biomaterials for implant placement. An incision similar to the first procedure was made over the tibial tuberosity. The grafted region was prepared for implant placement by applying a progressive sequence of drills (spear drill; 2.0 mm spiral drill - Neodent®; Curitiba, PR, Brazil) to accommodate a machined surface implant 4 mm high and 2.2 mm in diameter (Neodent®; Curitiba, PR, Brazil). All drilling was performed with the aid of an electric motor, adjusted to 1200 rpm, under abundant irrigation with sterile saline solution. The implant was installed with the aid of a digital key (1.2 mm hexagonal digital key - Neodent, Curitiba, PR, Brazil). The tissue suture and the postoperative drug protocol that was used were similar to those used in the first surgery.

At 15 and 45 days after the implant implantation surgical procedures, the animals were euthanized by an administration of a large anesthetic dose. The tibias were separated according to the performed analyses. The right tibia was used for microtomographic and histomorphometric analysis, whereas the left tibia was used for biomechanical analysis.

### Biomechanical Evaluation

After euthanasia, the left tibias were stabilized with a small splint. A hexagonal wrench was attached to both the implant and torque wrench (Tohnichi, model ATG24CN-S, Tokyo, Japan), and counterclockwise movement was performed to unscrew the implant. The maximum peak required to move the implant was noted as the removal torque value.

### Microtomographic evaluation

**Figure 1:**
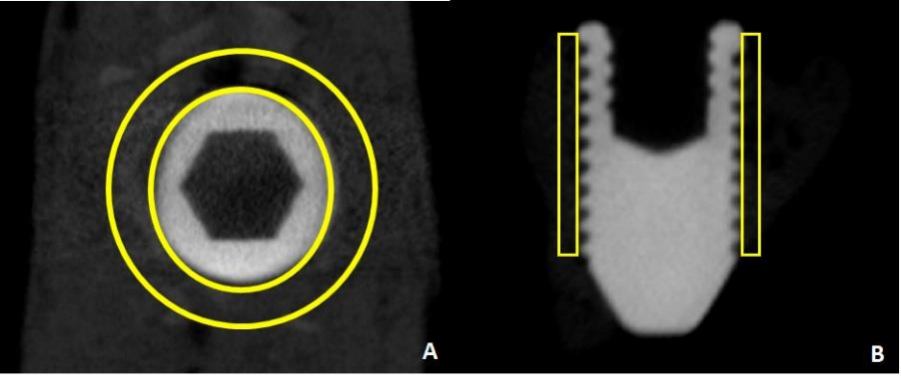
Scheme of the Micro CT analysis. A) Axial view of the region of interesting around the implants (Space between the yellow circles); B) Sagittal view of the region of interesting around the implants. Note that the evaluation was performed around the bony of the implants until de presence of the last thread (Space inside the yellow rectangles).

The right tibias were fixed in 4% paraformaldehyde for 48 hours and then stored in 70° alcohol. These samples were scanned by a microtomograph (Skyscan, Aatselaar, Belgium) with the following parameters: camera pixel, 12.45; X-ray tube power, 65 kVP; X-ray intensity, 385 µA; integration time, 300 ms; filter, Al-1 mm; and voxel size, 18 µm^3^. The images were reconstructed, spatially repositioned and analyzed by specific software (NRecon, Data Viewer, CTAnalyser, Aatselaar, Belgium). The region of interest (ROI) was defined as a 0.5 mm circular region around the entire diameter of the implant. This ROI was defined as the total volume (0.5 mm margin around implants - 4.5 mm x 3.2 mm). As the implants placed did not receive a cover screw in some cases, bone formation occurred inside the prosthetic platform. To prevent this bone formation from interfering with the analysis of the volume of mineralized tissue around the implant, a second ROI was defined to exclude the platform volume. With the results obtained in both ROIs, it was possible to define the bone formation volume using the formula Total volume - Platform volume = Volume of mineralized tissues (Figure 1). The threshold used in the analysis was 25-90 shades of gray and the volume values ​of mineralized tissue around the implants were obtained as a percentage. A trained examiner blinded to the experimental groups performed this analysis.

### Histomorphometric evaluation

After scanning, the right tibias were dehydrated in an increasing series of ethanols (60 - 100%), infiltrated and polymerized into light-curable resin (Technovit 7200 VLC, Kultzer Heraus GmbH & CO, Wehrheim, Germany). The blocks containing the implant and bone tissue were cut at a central point using a wear and tear system (Exakt Apparatebeau, Hamburg, Germany). The final sections were approximately 45-μm thick, stained with Stevenel's blue associated with acid fuchsin and analyzed under an optical microscope (DIASTAR - Leica Reichert & Jung products, Wetzlar, Germany) at 100X magnification. Histomorphometric evaluation was performed using image analysis software (ImageJ, San Rafael, CA, USA). The percentages of bone-implant contact (% BIC) and bone area between implant turns (% BBT) were evaluated separately in the first six implant casts. A blinded and trained examiner performed these analyses.

### Statistical analysis

GraphPad Prism 6 software (San Diego, CA, USA) was used to perform the statistical analysis in this study. The data generated by the histometric, microtomographic and biomechanical analyses were numerical; thus, these data were submitted to the Shapiro-Wilk normality test to evaluate if the data were distributed according to the central distribution theorem. Biomechanical data were not distributed according to normality, and the nonparametric Mann-Whitney test was used for the inferential analysis. Data from the microtomographic and histometric analyses presented a normal distribution, and then these data were analyzed using the parametric unpaired t-test. All tests in this study were conducted with a significance level of 95%.

## Results

All animals survived after the surgical procedures and were healthy throughout the experimental period. The sample size calculation was referenced to % BIC data from a previous study that evaluated the effect of implant surface osseointegration in grated areas in a similar experimental model and assessment as performed in this study ^13^. Considering that the smallest difference between the means in the groups in which there were statistically significant differences was 19.29% with a standard deviation difference between these groups of 6.59%, it was found that a sample of 7 animals per group/period was sufficient for using statistical tests with type α error set at 0.05 and β power of 0.90.

### Implants installed in DBB-grafted areas showed higher stability

Biomechanical analysis verified that implants placed in DBB-grafted areas presented greater removal countertorque values than implants placed in HA/TCP-grafted areas within 15 days (2.33 ± 0.51 Ncm vs. 1.50 ± 0.54 Ncm) (p <0.05) (Table 1).

**Table 1:** Mean and standard deviation of all the parameters tested in this study. * p <0.05; ** p < 0.01; *** p < 0.001- Higher values to the HA / TCP group over the 15-day period - Unpaired t-test

	Groups / Period	15 days	45 days
Removal torque (Ncm)	DBB	2.33 ± 0.51^*^	3.83 ± 1.16
	HA/TCP	1.50 ± 0.54	3.66 ± 1.86
BV/TV (%)	DBB	34.96 ± 3.68^**^	41.77 ± 6.06
	HA/TCP	25.61 ± 2.95	37.63 ± 3.47
%BIC	DBB	7.99 ± 6.22	20.87 ± 8.28^***^
	HA/TCP	7.36 ± 5.79	11.52 ± 7.42
%BBT	DBB	10.85 ± 9.94	26.83 ± 12.35^***^
	HA/TCP	13.24 ± 8.66	11.98 ± 7.56

### Implants installed in DBB-grafted areas showed a higher volume of mineralized tissues in the vicinity of implants

Microtomographic analysis verified that implants installed in DBB-grafted areas presented higher BV/TV values than implants installed in HA/TCP-grafted areas at the 15-day (34.96 ± 3.68 % vs. 25.61 ± 2.95 %) (p <0.01) (Table 1).

### Implants installed in DBB grafted areas showed a higher degree of osseointegration

**Figure 2:**
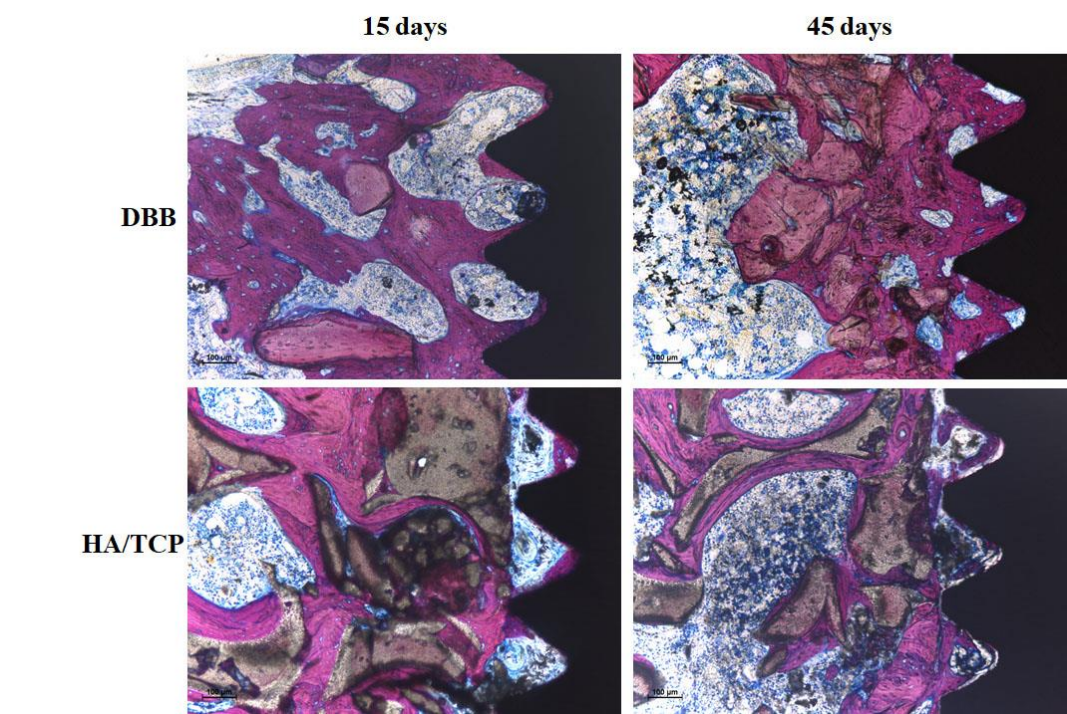
Representative images of the non-decalcified histological sections. It is possible to note the better pattern of osseointegration associated with the implants placed in the DBB-grafted area.

Histometric analysis verified that implants installed in DBB-grafted areas presented higher % (20.87 ± 8.28 % vs. 11.52 ± 7.42 %) and % BBT (26.83 ± 12.35 % vs. 11.98 ± 7.56 %) values ​​than implants installed in HA/TCP-grafted areas at the 45-day evaluation (p <0.001) (Table 1). Figure 2 shows representative images of the non-decalcified sections used to perform the histometric analysis.

## Discussion

In general, it was found in this study that implants installed in DBB-grafted areas presented higher values of removal torque and volume of mineralized tissues after 15 days and higher % BIC and % BBT at the 45-day evaluation compared with implants placed in grafted areas with HA/TCP. These findings may imply that the appropriate times for occlusal loading on implants placed in grafted areas with different osteoconductive biomaterials should be different, especially if the implants did not obtain the primary stability required for immediate loading. These outcomes conflicts with a finding of the previous pre-clinical study in a dog model that showed that the immediate and staged implants placed in areas grafted with DBB and HA/TCP presented the same level of osseointegration. However, the longer experimental period of evaluation (8 weeks) and the different pre-clinical models may be the reason of the differences of the outcomes of our study with this preview study ^15^.

Increased implant removal torque and mineralized tissue volume in earlier periods of osseointegration in DBB-grafted areas may be related to the lower degree of DBB resorption compared to that of HA/TCP; this lower degree of resorption is associated with higher osteoconductive properties of DBB. Indeed, a clinical study showed that the amount of DBB and HA/TCP in bone biopsies harvested after 5 months of the maxillary sinus lift procedure was 15.8 ± 2.1% in the HA/TCP group and 21.36 ± 4.83% in the DBB group ^11^. Another clinical study showed that biopsies removed from the maxillary sinus 180-240 days after the surgical procedure presented 26.6 ± 5.2% HA/TCP and 37.7 ± 8.5% ABB ^4^. These differences between these two osteoconductive bone substitutes in the resorption properties may be related to the mechanism of action of the biphasic ceramic since βTCP is resorbed whereas the HA portion maintained in the grafted area serves as a scaffold for bone formation ^16^ ^,^ ^17^. The lower reabsorption of DBB particles may lead to an increased volume of the grafted area, and this volumetric increase may have influenced the mechanical attachment of the implants in the grafted area.

Another interesting finding of this study was that the implants placed in areas grafted with DBB presented a higher degree of osseointegration observed at 45 days than the implants placed in HA/TCP-grafted areas. This fact may indicate that the distribution of new bone in the grafted area is even more important than the amount of the new bone, since the areas grafted with DBB presented less or an equal amount of bone formation than the areas grafted with HA/TCP in previous clinical studies ^4^ ^,^ ^18^ ^,^ ^19^. Indeed, one clinical study showed that DBB is more osteoconductive than HA/TCP since bone-to-graft contact was found to be significantly greater with DBB (48.2 ± 12.9%) than with HA/TCP (34.0 ± 14.0%). This finding can mean that implants can be subjected to occlusal load faster when they are placed in areas grafted with DBB compared with when implants are placed in areas grafted with HA/TCP.

Other factors may explain the superiority of osseointegration in implants in areas grafted with DBB. The experimental model used in this study was a noncritical defect made on the tibia that would promote the formation of bone in the grafted areas ^20^. Previous studies have shown that osteoconductive biomaterials show greater bone formation, greater osteoconduction, and smaller bone remnants when the bone substitute is close to the bone wall ^12^ ^,^ ^21^ ^,^ ^22^, which serves as a source of undifferentiated mesenchymal cells that will eventually become osteoblasts ^20^ ^,^ ^21^.

Despite the important findings of this study, it has some limitations that should be taken into account when analyzing the results. The use of machined implant surfaces is beneficial for purely assessing the effect of biomaterials on the osseointegration process; however, most clinical and preclinical studies evaluate the osseointegration process in graft areas with surface-modified implants ^12^ ^,^ ^14^. Therefore, the impact of different implant surface modifications on osseointegration in areas grafted with osteoconductive biomaterials should be further investigated. In addition, long-term evaluation of osseointegration in grafted areas is necessary to determine whether the observed results represent a real improvement in the pattern of osseointegration in DBB grafted areas or whether this phenomenon detected in our study was momentary and the osseointegration process in grafted areas with DBB and HA/TCP will be comparable at some point in the healing phase.

## Conclusion

It can be concluded that the implants placed in defects in areas grafted with DBB have a better osseointegration pattern than implants placed in defects in areas grafted with HA/TCP.
